# Investigating the Influence of Tacit Knowledge Transformation Approach on Students’ Learning Ability

**DOI:** 10.3389/fpsyg.2021.647729

**Published:** 2021-07-29

**Authors:** Liu Zheyu, Cui Weijin, Zhou Jihui, Wang Yuan, Usman Ghani, Xuesong Zhai

**Affiliations:** ^1^Faculty of Education, Tianjin Normal University, Tianjin, China; ^2^College of Education, Zhejiang University, Hangzhou, China; ^3^Department of Business Administration, Iqra University, Karachi, Pakistan

**Keywords:** tacit knowledge, transformation model, student well-being, online learning, social media

## Abstract

Tacit knowledge is an essential foundation for developing students’ learning ability, especially in understanding and solving problems. However, the transforming of tacit knowledge confront a big challenge during the outbreak of Corona Virus Disease 2019 (COVID-19), because most teaching and learning activities were conducted in online context, which impair a face-to-face interaction. To explore the effect of tacit knowledge on students’ learning ability in the online learning environments, the current study based on SECI model (The Socialization, Externalization, Combination, and Internalization) proposed to design the tacit knowledge transformation teaching approach. To assess the effectiveness of this approach, 60 elementary school students were recruited in the quasi-experiment. The results of retention test and transfer test showed that the experimental group, using the tacit knowledge transformation teaching approach, has significant improvement on learning ability than the control group. The current research theoretically provide a teaching strategy on tactic knowledge, and practically helps teachers to organize instructional activities, thereby, advocating the appropriate use of social media.

## Introduction

During the spread of Corona Virus Disease 2019 (COVID-19) globally, physical classes had to be transferred to online settings, which brought dramatic challenges to the traditional curriculum. Educators are getting adapted to online instruction (Chen et al., 2020). However, the online teaching setting is still facing many challenges, which may negatively affect the quality of learning and students’ well-being (Zandvliet et al., 2019). The reason may due to that online learning probably help enhance the comprehension and memorization of explicit knowledge, neglecting the tacit knowledge used to solve problems, and restricts teacher-student interaction.

Tacit knowledge considerably influences students’ ability to understand and solve problems in learning (Chamidy et al., 2020). Tacit knowledge was regarded playing a fundamental role in triggering problem discovery, and further facilitate contextual understanding (Collins, 2001). For example, the situation may commonly happen: students remembered how teachers solve problems but fail to figure them out in a new context. Ignoring the problem of transforming tacit knowledge leads students to be confused in problem solving (Chamidy et al., 2020), and may bring anxiety to students (Larcombe et al., 2013). Therefore, it is urgent to explore an implementable approach capable of transforming tacit knowledge effectively in online settings.

The Socialization, Externalization, Combination, and Internalization (SECI) model was defined as an organization which creates knowledge through the interactions between explicit knowledge and tacit knowledge. As a knowledge transformation approach proposed by Nonaka and Takeuchi (1995), the SECI approach has been widely accepted in the field of education. It illustrates the learning process at the level of knowledge transfer and transformation to enable students to explicitly and intentionally construct, update, and apply knowledge (Xue et al., 2020). The current study, based on SECI, proposed to construct a tacit knowledge transformation approach in online learning, and evaluate how the approach influences learners’ internalization of their tacit knowledge.

## Literature Review

### Learning Dilemmas Under the Epidemic

The outbreak of COVID-19 had serious implications on education worldwide. In response to the epidemic, schools opted to restore normalcy by employing conferencing software to deliver online teaching on a large scale (Adnan and Anwar, 2020). Despite these conference tools have improved the relevant functions to meet the needs of online teaching, some existing problems are still exposed. Owing to technical limitations, online education is generally instructor-centered on lecturing with a lack of interaction between teachers and students (Chen et al., 2020). As a result, it leads not only to deficiency of translation and application of knowledge in problem solving, but also to lack of interpersonal communication and interaction in the instructional system (Adnan and Anwar, 2020).

### Tacit Knowledge

Knowledge is typically classified into tacit knowledge and explicit knowledge (Polanyi, 1962). Different from the explicit knowledge, tacit knowledge has been defined as personal knowledge that is difficult to formalize and describe through language (Tamzini, 2015). Tacit knowledge is more fundamental than explicit knowledge. It was found that students with high tacit knowledge gained higher academic achievement than those with low tacit knowledge (Chamidy et al., 2020). Besides, tacit knowledge as an experience and skill promote student understanding and solving problems (Ibidunni et al., 2018). Prior research has substantiated that the effective transformation of tacit knowledge may improve students’ ability to comprehend and solve problems, thereby enhancing their sustainable well-being (Tan et al., 2019).

### Tacit Knowledge Transformation Approach

The SECI model consists of four knowledge transformation modes: SECI (Nonaka et al., 2000). Students concentrate solely on the explicit knowledge in the book or the teacher’s language, making it difficult for them to develop the necessary associations, imaginations, and deductive thinking. Through the construction of a tacit knowledge transformation approach, researchers can design student-student interactions that promote a positive classroom climate (Nurmi and Kiuru, 2015). It can also guide tacit knowledge transformation to achieve a higher level of students’ problem understanding and solving (Chamidy et al., 2020).

The implicit nature of tacit knowledge makes it difficult for learners to access it directly through explicit symbols, which compromises the activation, transformation, and construction of tacit knowledge in the classroom (Ambrosini and Bowman, 2001). The successful experience of tacit knowledge transformation in the field of management is based on tacit knowledge transformation approach (Sasaki, 2017). Existing research on tacit knowledge transformation in online learning emphasizes the use of a specific technique or strategy (Silby and Watts, 2015). Hence, the design and development of tacit knowledge transformation approaches in online environment settings require further research.

## Method

### Designing

**FIGURE 1:**
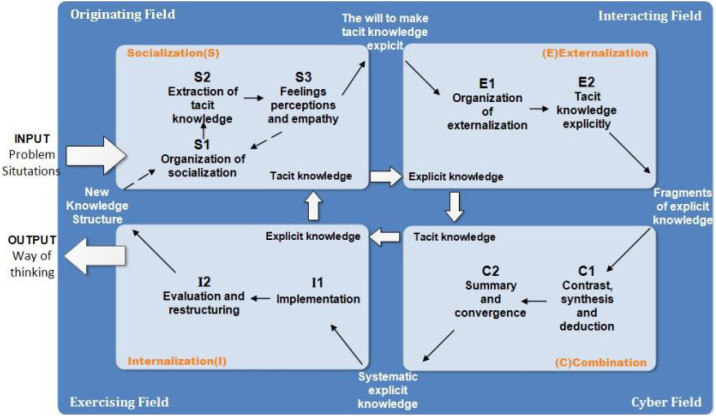
The approach of tacit knowledge transformation in online classroom.

The present study builds a tacit knowledge transformation approach for online learning (see Figure 1) based on the SECI model. The tacit knowledge transformation approach in online classroom focuses on the spiral from tacit knowledge to explicit knowledge fragments, combinatorial explicit knowledge, and internalization of tacit knowledge. The spiral process leads to the generation of higher-order ways of thinking. To achieve this, originating, interacting, cyber, and exercising fields were constructed with technological support (Nonaka et al., 2000), thus aiding the transformation from socialization (organization of socialization, extraction of tacit knowledge, and feelings perceptions and empathy) to externalization (organization of externalization and tacit knowledge explicitly), to combination (contrast, synthesis and deduction and, summary and convergence), and to internalization (implementation and, evaluation and restructuring). The approach assists in creating the context by supporting the “field” (i.e., originating field, interacting field, and cyber field and exercising field) environment with information technology.

The originating field is a place of tacit knowledge exchange among the learners, providing an original basis for the learner to share their experiences and helping them to socialize with each others. The transformation of tacit knowledge at this stage can be implemented through observational learning, activity-based learning, and symbolic learning. For example, teachers may set up scenario-based case observation activities in the virtual classroom online or narrative activities among students. Social Networking Services (SNS) platforms (Jeong et al., 2015), online communities (Kassem et al., 2015), and social media interactions (Chatti et al., 2007) can effectively assist in the construction of the original field and the convergence of tacit knowledge.

The interacting field is a place where knowledge activities took place, providing contextual support for an open presentation and deep communication among learners. In the externalization stage, learners collect and organize materials, and teachers guide students to articulate and communicate their tacit knowledge with others. Learners share their tacit knowledge through metaphors, simulations, or diagrams, transforming it into explicit knowledge fragments that could spread on the social media. Online groups (Thomas and Thorpe, 2019) and multi-device platforms (Little and Knihova, 2014) can support learners’ knowledge externalization in addition to media interaction.

The cyber field is a place where the pieces of explicit knowledge generated in the previous stage, are combined and processed, and new knowledge is generated. Knowledge is systematized in the learning community through comparison, synthesis, deduction, and reasoning. In the combination stage, collaboration between learning committees play an important role. They collaborate to summarize the fragmented explicit knowledge to form the systematic explicit knowledge in the learning community. The construction of system fields can be effectively supported by cognitive map (Rodhain, 1999) and mind map (Leckie et al., 2010), which help in the combination of explicit knowledge.

The exercising field is a place where new knowledge is applied, evaluated, and reflected on in practical exercises. In the internalization stage, the explicit knowledge of the community is internalized into the conscious behavior of the students through practice. After the practice, the students will assess and reconstruct their tacit knowledge structures and thinking patterns. Educational games (Noemí and Máximo, 2014) and virtual reality (Smith et al., 2012) effectively create real-life situations and help learners to learn by practice.

### Participants

The participants were recruited from an elementary school in Eastern China. We collected valid data from 60 sixth-grade students, of which 32 were male and 28 were female, 31 students age was ranged from 10 to 12 years, while 29 students age was ranged from 12 to 14 years, 35 students belong to urban area and 25 students belong to rural area (see Table 1). The participants were randomly divided into two groups (control group and experimental group) with 76 subjects in each group. A gift was offered to all the respondents for their participation.

**TABLE 1:** Demographic variables (*N* = 60).

**Variables**	**Category**	***N***	**Percentage (%)**
Gender	Male	32	53.33
	Female	28	46.67
Age	10–12	31	51.67
	12–14	29	48.33
Residence	Urban	35	58.33
	Rural	25	61.67

### Procedure

The pre-test of the control and experimental classes were two math test scores (midterm test and September joint test). Each participant received an instruction sheet and a consent form before the experiment.

During the experiment, the online application DingTalk is used as a teaching platform. First, the teacher completes the instructional design of the “Understanding the Circle” on his/her own and teaches it in the control group. After completing the lesson, the instructors ask students to fill in the post-test which is a learning outcome test. The test of learning outcomes is a standardized paper that contained a retention test and a transfer test. Retention-transfer test is widely used both domestically and internationally (Cheng and Beal, 2020; Knoop-van Campen et al., 2020). Secondly, the teacher teaches in the experimental class based on the teaching design of “Understanding the Circle” developed by the SECI model, and teaches it in the experimental group. After completing the lesson, we ask students to fill out the learning outcome tests. Finally, took back the test questions for grading. Concrete teaching details and items of the learning outcome test are available in the supplementary files.

## Results

The SPSS 21.0 was employed in analyzing the collecting data. The descriptive results of pre-test and post-test scores for two groups are shown in Table 2. To check the normality of the data, Kline’s (2015) criteria was used, which states that the data are normally distributed when the skewness and kurtosis are under | 3| and | 10| respectively. The final results for skewness (−1.062 to 1.516) and kurtosis (−1.132 to 3.854) values indicate that both the pre-test, retention test, and transfer test were normally distributed.

**TABLE 2:** Descriptive statistical data of the two groups’ pretest and posttest.

**Test**	**Group**	***N***	**Mean**	**SD**
Pretest	Experimental group	30	83.70	8.53
	Control group	30	85.40	9.89
Posttest for the retention of knowledge	Experimental group	30	89.38	14.42
	Control group	30	67.08	18.42
Posttest for the knowledge transfer	Experimental group	30	73.33	20.69
	Control group	30	37.08	18.12

Therefore, the ANCOVA test was employed to analyze the difference in retention and transfer performance between the two groups. Regarding the retention of knowledge (see Table 3), there was significant difference (*F* = 311.41, *p* < 0.0001) between the two groups, showing that the SECI model was able to improve student retention performance. The analysis of the knowledge transfer test (see Table 4) showed a significant difference (*F* = 323,13, *p* < 0.0001) between two groups, which indicated that students’ knowledge transfer was enhanced with the utilization of the SECI model.

**TABLE 3:** ANCOVA of the posttest for the retention of knowledge.

**Group**	***N***	**Mean**	**SD**	**Adjusted mean**	***F***	**η2**
Experimental group	30	89.37	14.41	90.81	311.41***	0.85
Control group	30	67.08	18.77	65.65		

***p < 0.001.

**TABLE 4:** ANCOVA of the posttest for the knowledge transfer.

**Group**	***N***	**Mean**	**SD**	**Adjusted mean**	***F***	**η2**
Experimental group	30	73.33	20.69	74.94	323.13***	0.85
Control group	30	37.08	18.12	35.47		

***p < 0.001.

## Discussion

The current study investigates the underlying mechanism of transformation of tacit knowledge to promote students’ learning ability in understanding and solving problems. An approach of tacit knowledge transformation for online learning is designed in this study.

Before the approach was implemented, the teacher reported some reflections on tacit knowledge transformation:

*Although I pay more attention to the students’ understanding, considering the low motivation of the students and the lack of effective means of transformation, when doing some exercises, even if it has some implicit context, we often ignore it, that is, to directly practice their calculations. I think it would be helpful if some implementable online tacit knowledge transformation approach could be provided to teachers.*

In this excerpt, the teachers reflectively analyzed their ideas in the transformation of tacit knowledge. They argued that the dilemma of transforming tacit knowledge in online environment is widespread, and the central problem is the lack of effective instructional means. This offers useful insights, as teachers recognize that effective approaches of tacit knowledge transformation are essential. Likewise, Songkram and Chootongchai’s (2020) study indicates that using the SECI model to share knowledge in the classroom allows students to develop their creative potential. Therefore, this study, further examined the performance of students’ learning with and without the tacit knowledge transformation approach.

After the experiment, the results confirmed that the tacit knowledge transformation approach with four stages (socialization, internalization, externalization, and combination) were successful, indicating that the tacit knowledge transformation approach promoted students’ cognitive development at all stages. Existing research has identified socialization (transformation of tacit knowledge to other forms of tacit knowledge) and externalization (transformation of tacit knowledge to explicit knowledge) as important factors influencing students’ learning outcomes and learning experience (Ibidunni et al., 2020). Without significant differences between students’ levels, there were significant differences in both retention and transfer scores of students after the experiment. Among this, transfer ability showed the most significant difference which indicates that the transformation approach of tacit knowledge can effectively contribute to students’ ability to understand and solve problems. Through the use of this approach, the teacher made the following observations:

*I think this approach can be very well applied to the online classroom. I feel that I was able to use this approach very well and my students’ thinking was able to be developed through it. The core idea of mathematics is to develop students’ thinking level, so I will change my teaching method in the future.*

*I have noticed that students that used the tacit knowledge transformation approach can transform their tacit knowledge into mind maps and apply it consciously in their practice. Each student communicates what they have learned in their group, completing the mind map and then presenting it to the whole class. This process helped them to improve their problem solving skills.*

In these narratives, teachers demonstrated an encouraging view about tacit knowledge transformation approach that could successfully assist students in understanding and solving problems. The transformation of tacit knowledge is carried out through instructional activities. There are three types of instructional activities of interactions in class: between teacher and student, between student and student, and by students themselves. By using the tacit knowledge transformation approach, teachers were able to solve the confusion of students whose tacit knowledge could not be transformed and paved the cognitive level building blocks to enhance students’ well-being.

## Limitations and Future Study

The current study is not lacking limitations and should be acknowledged by future research. First, the number of participants were small, which may limit the generalizability of the results. Future research should investigate whether the approach for transformation of tacit knowledge have the same impact on larger number of students, so that our findings could be validated further. Second, this study only interviewed teachers and did not collect feedback from students. Future research should could incorporate students’ emotional experiences for further analysis, such as interviews, FaceReader. Third, this study only included a limited number of teachers in the interviews, thus their comments may not be representative. We suggest that future research could interview more teachers and conduct a discourse analysis of their comments.

## Data Availability Statement

The raw data supporting the conclusions of this article will be made available by the authors, without undue reservation.

## Ethics Statement

The studies involving human participants were reviewed and approved by the Ethics Committee of Tianjin Normal University, China. Written informed consent to participate in this study was provided by the participants’ legal guardian/next of kin.

## Author Contributions

LZ and XZ: conceptualization. CW: formal analysis and writing—original draft preparation. ZJ and WY: investigation. LZ, UG, and XZ: writing—review. All authors have read and agreed to the published version of the manuscript.

## Conflict of Interest

The authors declare that the research was conducted in the absence of any commercial or financial relationships that could be construed as a potential conflict of interest.

## Publisher’s Note

All claims expressed in this article are solely those of the authors and do not necessarily represent those of their affiliated organizations, or those of the publisher, the editors and the reviewers. Any product that may be evaluated in this article, or claim that may be made by its manufacturer, is not guaranteed or endorsed by the publisher.
